# A neural network based holistic model of ant route navigation

**DOI:** 10.1186/1471-2202-13-S1-O1

**Published:** 2012-07-16

**Authors:** Bart Baddeley, Paul Graham, Philip Husbands, Andrew Philippides

**Affiliations:** 1 Centre for Computational Neuroscience and Robotics, Department of Informatics, University of Sussex, Brighton, BN1 9QG, UK; 2 Centre for Computational Neuroscience and Robotics, School of Life Sciences, University of Sussex, Brighton, BN1 9QJ, UK

## 

The impressive ability of social insects to learn long foraging routes guided by visual information [1] provides proof that robust spatial behaviour can be produced with limited neural resources [2,3]. As such, social insects have become an important model system for understanding the minimal cognitive requirements for navigation [1]. This is a goal shared by biomimetic engineers and those studying animal cognition using a bottom-up approach to the understanding of natural intelligence [4]. Models of visual navigation that have been successful in replicating place homing are dominated by snapshot-type models where a single view of the world as memorized from the goal location is compared to the current view in order to drive a search for the goal [5], for review, see [6]. Snapshot approaches only allow for navigation in the immediate vicinity of the goal however, and do not achieve robust route navigation over longer distances [7].

Here we present a parsimonious model of visually guided route learning that addresses this issue [8]. We test this proposed route navigation strategy in simulation, by learning a series of routes through visually cluttered environments consisting of objects that are only distinguishable as silhouettes against the sky. Our navigation algorithm consists of two phases. The ant first traverses the route using a combination of path integration and obstacle avoidance during which the views used to learn the route are experienced. Subsequently, the ant navigates by visually scanning the world – a behaviour observed in ants in the field – and moving in the direction which is deemed most familiar. As proof of concept, we first determine view familiarity by exhaustive comparison with the set of views experienced during training. In subsequent experiments we train an artificial neural network to perform familiarity discrimination using the training views via the InfoMax algorithm [9].

By utilising the interaction of sensori-motor constraints and observed innate behaviours we show that it is possible to produce robust behaviour using a learnt holistic representation of a route. Furthermore, we show that the model captures the known properties of route navigation in desert ants. These include the ability to learn a route after a single training run and the ability to learn multiple idiosyncratic routes to a single goal. Importantly, navigation is independent of odometric or compass information, does not specify when or what to learn nor separate the routes into sequences of waypoints, so providing proof of concept that route navigation can be achieved without these elements. The algorithm also exhibits both place-search and route navigation with the same mechanism. As such, we believe the model represents the only detailed and complete model of insect route guidance to date.
